# ADIBAS-Volterra aircraft trajectory prediction model based on improved dynamic integration of beetle tentacle searching

**DOI:** 10.1371/journal.pone.0323718

**Published:** 2025-06-02

**Authors:** Lin Li, Shiyan Sun, Huimin Zhu, Chaobing Zheng, Yaqin Zeng

**Affiliations:** 1 Department of Weaponry Engineering, Naval University of Engineering, Wuhan, China; 2 The School of Information Science and Engineering, Wuhan University of Science and Technology, Wuhan, China; Federal University of Technology - Parana, BRAZIL

## Abstract

The prediction of aircraft manoeuvre trajectories is an important prerequisite for decision making. However, how to achieve real-time and scientific aircraft manoeuvre trajectory prediction using trajectory data needs to be addressed urgently. To solve this problem, we propose a hybrid algorithm based on Improved Beetle Antennae Search (BAS), Aircraft Manoeuvre Boundary Point Identification algorithm, Adaptive Dynamic Integration (ADI) and Volterra series, called ADIBAS-Volterra. Firstly, a large amount of trajectory sample data is trained to construct the BAS-Volterra algorithm suitable for predicting aircraft manoeuvre trajectories, which achieves a balance between global and local solutions. Secondly, in order to improve the accuracy of the online manoeuvre trajectory prediction of our proposed model in complex environments, the parameters of the whole prediction model based on the BAS-Volterra algorithm are adaptively updated according to the identification results of the aircraft manoeuvre boundary points, including the optimisation of the algorithmic weights and the optimisation of the parameters. Compared with the existing state-of-the-art methods, the newly proposed aircraft manoeuvre trajectory prediction algorithm adopts K-means clustering to initialise the tentacle position, which can flexibly adjust the search strategy at different stages and make the algorithm more reasonable. Four measures, Relative Root Mean Square Error (RRMSE), Mean Absolute Deviation (MAD), Mean Absolute Percentage Error (MAPE) and Normalised Mean Square Error (NMSE) were used to assess prediction accuracy. Finally, the scientific validity of the proposed algorithm is verified using Mackey Glass and Rossler datasets.

## Introduction

Trajectory prediction is to make scientific prediction results for the future trajectory of the aircraft according to the important information contained in the collected trajectory, and precise prediction of the aircraft manoeuvre trajectory provides a key foundation for the assessment of the destructive effect and decision-making, so the study of aircraft manoeuvre trajectory prediction problem has important practical significance and application value.

Recently, research on aircraft manoeuvre trajectory prediction can be divided into two main directions: model-based approaches and data-based approaches. Model-driven methods are based on physical motion models and kinematic principles, combined with the dynamic characteristics of the aircraft and environmental influences to build mathematical-physical models, Finally, accurate prediction of the future trajectory of the aircraft is achieved. T. Baklacioglu [1] proposed a flight trajectory prediction model for a aircraft in the climb and dive phases based on genetic algorithms. Zhang *et al*. [2] aimed to develop deep learning models for trajectory prediction by developing a deep learning model to improve the safety of en-route flights, in which the uncertainty of the model prediction is characterized using Bayesian methods. Wang *et al*. [3] proposed an extended Kalman filter trajectory prediction algorithm based on constant angular velocity and velocity kinematics models, which can effectively predict the future trajectory of the aircraft. Zhang *et al*. [4] focused on the study of trajectory prediction algorithms and their error analysis in trajectory prediction. We collected a large number of flight trajectory data as samples, processed the sample data using GIS, built a flight motion model based on the data, and predicted the sample data using the Kalman filter algorithm.

The aircraft manoeuvre trajectory prediction method based on the data-driven approach takes the neural network as the core and combines a great quantity of data to build the aircraft manoeuvre trajectory prediction model. The research on data-driven aircraft manoeuvre trajectory prediction methods is generally regarded as a time series prediction problem [5]. Xi *et al*. [6] proposed an air combat aircraft manoeuvre identification model based on an online ensemble semi-supervised classification framework with online learning, ensemble learning, semi-supervised learning and tri-training algorithms, which improves the accuracy and adaptability under highly dynamic air combat conditions. Compared to land traffic, sparse waypoints and shared flight paths make flight trajectory prediction difficult, so Shi *et al*. [7] proposed a constrained long- and short-term memory network for flight trajectory prediction. Based on the dynamic characteristics of the aircraft, three constraints were proposed for the climb, cruise and descent/approach phases, namely climb altitude, waypoints and runway direction, and the model was able to maintain long-term dependencies through dynamic physical constraints. To improve the optimization performance of Differential Evolutionary Algorithm (DE), Zhong *et al*. [8] proposed a hybrid optimization trajectory prediction algorithm based on Differential Evolutionary Algorithm (DE) and Harris Hawk Optimization Algorithm (HHOA), which outperforms the other methods under the same conditions. Aircraft manoeuvre trajectory prediction is a time series prediction problem, the method does not require an accurate aircraft motion model, the method has a simple structure, but the parameter setting process of the model is more complicated, as well as the sample size of the data is very demanding.

Time series based aircraft motion trajectory prediction methods also include Volterra model [9–11], Gaussian mixture model [12], fuzzy time series prediction [13], DNN [14], CNN [15], echo state neural network [16], LSTM [17–19]. The parameters of LSTM [5] are optimized and improved using gradients to collect the position, attitude and posture information of the aircraft so as to achieve accurate prediction of the aircraft manoeuvre trajectory. Shi *et al*. [20] constructed some new dimensional features based on existing historical trajectory data. By analysing the statistical features of the three-dimensional data of longitude, latitude and altitude, a combined prediction model based on LSTM and ARIMA is proposed, and this combined model improves the accuracy of trajectory prediction.

Volterra [21] is a method for describing nonlinear systems, revealing the nonlinear characteristics of the system and the effect of time delays on the output, and is widely used in trajectory prediction. The complexity of the electromagnetic environment, the accuracy of the detection equipment and anti-jamming limitations, resulting in the trajectory prediction of the accuracy and robustness of the trajectory is not high, based on the aircraft information acquired by the sensor, combined with the Beetle Antenna Search algorithm and online learning algorithms based on the BAS Adaptive Dynamic Integration (ADI) of the ADIBAS-Volterra aircraft trajectory prediction model, the innovations of the model are as follows:

(i) The strategy of Adaptive Reconstruction Method (ARM) to maintain stability while adapting to new data can be elaborated through the following aspects: (1) The core of the Adaptive Reconstruction Method (ARM) lies in its dynamic adjustment mechanism. The mechanism relies on real-time monitoring and data collection to assess the effectiveness of the model and reveal potential problems by analyzing its current performance. This dynamic feedback allows the model to identify new data patterns in a timely manner, while reducing over-reliance on historical data. (2) During the adaptive reconstruction process, the system monitors the input data and model outputs in real time and uses statistical methods to assess the performance of the current model. This process includes the analysis of model prediction errors and feature screening by setting thresholds to ensure that only features that have a significant impact on the prediction results are retained. This approach can effectively prevent the negative impact on model performance due to noise or non-scalar features introduced by new data. (3) Adaptive reconstruction methods perform feature selection and reconstruction through intelligent algorithms such as genetic algorithms and particle swarm optimization. Whenever new data is input, the input features are dynamically updated according to the current prediction error, and only those features with strong correlation with the target output are retained, thus reducing the complexity of the model, avoiding the overfitting problem caused by feature redundancy to a certain extent, and improving the stability of the model. (4) The adaptive reconstruction method introduces a multi-level feedback loop. In the process of model adjustment, a dynamic weight allocation mechanism is formed through the cumulative analysis of historical prediction errors. Different historical data are given different historical window sizes and attenuation factors to ensure that the model’s memory of historical data is not too strong when new data arrives, so as to maintain the ability to adapt to new data. (5) In the parameter update of the model, ARM utilizes the adaptive learning rate. This approach allows the model to update its parameters with different strengths when adjusting them according to the magnitude of fluctuations in the current prediction error. Specifically, when the model performs poorly, the adaptive learning rate allows for more aggressive parameter adjustments; whereas, when the model performs stably, the reduction in the learning rate effectively prevents the model from being over-trained and maintains the stability and reliability of the model. (6) ARM combines a variety of intelligent algorithms to make the model have good adaptability. Through the adjustment of the mathematical model and its parameters in the dynamic environment, the changing environmental conditions can be instantly fed back into the model, ensuring that the prediction process is adjusted in time with the changes in the environment, thus improving stability. (7) The adaptive reconstruction strategy can improve the robustness of the model in complex and dynamic environments. When dealing with real-time data, it ensures that the prediction results are real-time and accurate through rapid feature reorganization and parameter optimization, so as to meet the demands of real-time applications while ensuring stability.

In summary, the adaptive reconstruction method effectively maintains the stability of the model while adapting to new data through the mechanisms of dynamic adjustment, real-time monitoring, feature selection, multi-level feedback, adaptive learning rate, and high adaptability to the environment. This approach enables the ADIBAS-Volterra model to consistently provide reliable prediction results under complex dynamic environments.

(ii) The improved Beetle Antenna Search Algorithm (BAS) takes the following factors into account when designing the multiscale search strategy:(1) Stochastic search capability: in order to enhance the global exploration capability of the search algorithm, a temperature parameter is introduced into the improved BAS. This parameter is used to regulate the magnitude of random perturbations during the search process. During the high temperature phase of the search, the algorithm tends to perform larger random perturbations, thus enhancing the randomness and exploration ability of the search. This strategy helps the algorithm to jump out of the local optimal solution and increase the likelihood of finding the global optimal solution. (2) Adaptive learning rate: the improved BAS algorithm uses an adaptive learning rate to update the tentacle positions. The design of adaptive learning rate makes it possible to flexibly adjust the search strategy at different stages and dynamically optimize the learning rate based on the current search state, which can maintain a better balance in the search process and prevent over-adjustment or slow convergence. (3) Tip position updating strategy: In the updating of tip positions, the algorithm considers the combination of the current optimal tip position and the random perturbation factor, so as to achieve a more efficient search. This updating strategy can quickly adjust the tentacle position according to the existing information to ensure the stability of the search process. (4) Multi-scale search strategy: the improved BAS enters into different search phases, combining the characteristics of other algorithms and adjusting at different scales. This multi-scale search strategy enables the algorithm to explore in a larger range, while also performing a refined search in the local area, so as to effectively deal with the changes of the objective function in the multi-dimensional space. (5) Dynamic influence of the surrounding environment: the improved BAS takes into account the dynamic change factors of the external environment, and adjusts the search strategy at the right time by real-time monitoring of the changes in the objective function, which makes the algorithm more flexible to deal with complex environments and unexpected situations.

In summary, the improved BAS algorithm fully considers the stochastic search capability, learning capability and environmental adaptability by introducing the temperature parameter, adaptive learning rate, dynamic updating of the tentacle position and multi-scale search strategy, thus achieving higher prediction accuracy and real-time performance in the target manoeuvre trajectory prediction model.

The BAS has a strong global optimization ability, can effectively avoid falling into local optimal solutions, and can flexibly adjust the search strategy in different search environments, which improves the search efficiency, but when dealing with large-scale problems, the convergence speed may be slower, and more iterations are needed to achieve satisfactory results, and the performance of the algorithm is more sensitive to the selection of parameters, and more experiments are needed to optimize the parameters.

The BAS-Volterra prediction model inherits the basic idea of sequence learning of online prediction model, and the BAS algorithm in the process of online learning uses K- means clustering for the tentacle position initialization, combined with an adaptive learning rate to cope with the variation of the objective function in multi-dimensional space. At the same time, the stochastic exploration ability of the algorithm is enhanced by introducing temperature parameters, which can flexibly adjust the search strategy at different stages, so as to achieve an effective balance between global and local solutions. The algorithm demonstrates good stability. In addition, considering the advantage of ensemble learning in improving model accuracy, ensemble learning is further introduced on the basis of BAS- Volterra trajectory prediction algorithm. According to the manoeuvring characteristics of the aircraft, the model parameters and weights are adaptively updated, and a new ADIBAS-Volterra prediction model is proposed, which constructs a suitable framework for aircraft manoeuvring trajectory prediction and improves the accuracy of aircraft manoeuvring trajectory prediction.

## Background techniques

### Beetle tentacle search algorithm

BAS [22] is an optimization algorithm based on the foraging behavior of beetles in nature, which constructs a multi-dimensional search space to find the optimal solution by simulating the tentacle detection mechanism of beetles in the process of finding food. First, the algorithm randomly initializes a group of ’beetles’ representing different solutions, and then these individuals explore the search space by adjusting their positions and speeds. During this process, each beetle updates its position based on its own experience and information from its peers, thus continuously moving closer to a better solution. When a beetle discovers a more optimal solution, it communicates this information to other beetles, encouraging the whole group to move towards the more optimal solution. This collective intelligence effectively prevents the algorithm from falling into local optimality and increases the probability of finding the global optimal solution. The basic steps of BAS include initialization, position updating, information transfer and termination condition judgement, and it is suitable for solving complex problems such as function optimization, combinatorial optimization and constrained optimization. Its advantages lie in its ability to adaptively adjust the search strategy, its strong global search capability, and its simple implementation, which has shown good practical application results [22].

#### Adaptive reconstruction method

Adaptive Reconstruction Method (ARM) [23] is a theory and methodology for effectively solving complex system analysis and optimization problems by dynamically adjusting the model structure and parameters to improve system performance and responsiveness. At its core is the dynamic adaptation mechanism: the system analyses current model performance through real-time monitoring and data collection to assess its effectiveness and identify potential problems. This process includes model updating, feature selection, data processing, etc., so that the system can better respond to complex environments and sudden changes. The key to adaptive reconfiguration is its adaptive capability, enhanced by intelligent algorithms such as genetic algorithms, particle swarm optimization or fuzzy logic to increase flexibility in dynamic or complex environments. The operational steps include data acquisition and analysis, model evaluation, reconstruction strategy development and model updating, forming a feedback loop for continuous optimization. In summary, the adaptive reconfiguration method is an important tool in modern scientific research and engineering practice by deeply analysing the operational data and adjusting the model in real time to adapt to the changing environment [24].

#### Manoeuvre trajectory prediction model based on improved BAS-Volterra algorithm

The BAS-Volterra algorithm is designed to predict the trajectory of a aircraft in real time by using the ship’s detection equipment to transmit the aircraft’s status information (speed, altitude, azimuth and pitch) and the ship’s status information (position, heading and speed) and environmental factors wind speed, wind direction, detection equipment (noise mean, noise standard deviation), electronic interference (wavelength, frequency, period, signal strength, pulse width of interfering signals).

On the one hand, when facing the threat of the aircraft, the ship can receive the status information of the aircraft in real time, including speed, altitude, azimuth and pitch angle. In addition, the ship’s own status information, such as position, heading and speed, is also important for trajectory prediction and environmental factors. Second, in building the BAS-Volterra model, the adaptive reconstruction method optimizes the input data and streamlines the data matrix used in the model by filtering out the features Strong impact on forecasting results, thus improving the computational efficiency and accuracy when dealing with real-time data. Thirdly, the use of Volterra level modelling is able to deal with the complex non-linear relationship between the aircraft and ship states. The trajectory of the aircraft is affected not only by the law of its own motion, but also by a variety of factors such as environmental factors, ship state, etc. With the introduction of higher-order time series functions, the Volterra model can deeply explore these complex relationships to achieve higher accuracy of trajectory prediction. Fourth, the improved BAS algorithm is used to optimize the kernel coefficients, which enables the model to quickly converge to the optimal parameter configuration through continuous iterative evaluation. This rapid adaptability allows the prediction model to be adjusted in a timely manner to accommodate dynamic changes in the state of the aircraft and ship in an ever-changing tactical environment. Fifth, according to the BAS-Volterra time series prediction algorithm, it is possible to take quick actions in complex environments. In summary, we propose an aircraft manoeuvre trajectory prediction model that incorporates the improved BAS-Volterra.

#### Time series forecasting modelling process with the BAS-Volterra algorithm

To improve the prediction accuracy and computational efficiency of the Volterra time series model, we introduce the improved beetle tentacle search algorithm into the Volterra model. The beetle tentacle search algorithm, by simulating the foraging behaviour of beetles in nature, has a global search capability that can effectively avoid the local optimal solution, thus optimizing the parameter settings of the Volterra model and accurately capturing the non-linear and time-varying characteristics in the time series. The simultaneous optimization process can adjust the model parameters in real time, making the model more adaptive and robust, and the prediction results more reliable, especially when dealing with complex data with large noise in the aircraft trajectory.

Firstly, the trajectory prediction system is set up by inputting the dynamic state parameters of the aircraft and the vessel, which include speed, altitude, azimuth, pitch angle and position information, while the state of the vessel includes position, heading, speed and environmental factors including wind speed and direction detection equipment (noise mean, noise standard deviation) electronic interference (wavelength, frequency, period, signal strength, pulse width of interfering signals). Secondly, the input features are screened according to the preset performance metrics, and by calculating the prediction error and comparing it with the set threshold, the features that have a great influence on the prediction results are retained, and the input features are adjusted based on the current prediction error to improve the predictive capability of the model. Again, Volterra-level modelling effectively captures the non-linear dynamic behavior of the target, thus further improving the scientific validity of the model. Meanwhile, the kernel coefficients are optimized using an improved beetle tentacle search algorithm to find the best parameter configurations and minimize the loss function of the model. Finally, the optimized kernel coefficient matrix is used to predict the aircraft trajectory, and the predicted aircraft trajectory is finally output, the steps are as follows.

(1) Inputting shipboard detection parameters, the model involves the following aircraft and ship dynamic state parameters [25]:

aircraft dynamic state parameters:

Velocity $v_{d}(t)$: The flight velocity of the aircraft at the *t* moment, in m/s.

Altitude *h*_*d*_(*t*): altitude of the aircraft at the *t* moment, in m.

Azimuth $\theta_{d}(t)$: azimuth of the aircraft at the *t* moment, unit is degree (^∘^).

Pitch angle $\phi_{d}(t)$: Pitch angle of the aircraft at *t* moment in degrees (^∘^).

Position (dimension *lat*_*d*_(*t*), longitude *lon*_*d*_(*t*)): the geographical position of the aircraft at the *t* moment in degrees (^∘^).

Vessel dynamic state parameters:

Position (Dimension *lat*_*b*_(*t*), Longitude *lon*_*b*_(*t*)): the position of the ship at moment *t* in degrees (^∘^).

Heading $\psi_{b}$: the ship’s heading angle at time *t*, in degrees (^∘^).

Speed $v_{b}$: the speed of the ship at the moment *t*, unit is m/s.

Heading Angle of Ships $\psi_{b}(t)$: $\psi_{s}(t)=tan^{-1}\left(\frac{lan_{d}(t)-lat_{b}}{lon_{d}(t)-lon_{b}}\right)$

Aircraft pitch angle $\phi_{d}(t)$: $\phi_{d}(t)=tan^{-1}\left(\frac{h_{d}-h_{b}}{d}\right)$

Yaw angle of aircraft $\psi_{d}(t)$: $\psi_{d}(t)=\theta_{d}(t)-\psi_{b}(t)$

where *h*_*d*_ is the height of the aircraft and is the height of the ship set to zero.

Environmental factor parameters:

Wind speed $v_{w}(t)$: the speed of the wind at time t, in m/s, which affects the trajectory of the object.

Wind direction $\alpha_{w}(t)$: the direction of the wind at time *t* in degrees.

Sensor noise *n*(*t*): the noise generated by the sensor, commonly used parameters include

Noise average *a*_*n*_: usually set to 0 (representing the ideal state), in practice it may be a constant average.

Noise Standard Deviation $\sigma_{n}$: The standard deviation of the measured value, a common quantification of measurement uncertainty.

Electronic Interference Degree *e*(*t*): A numerical value describing the effect of possible electronic interference on the object navigation system, commonly used parameters include

Interference Wavelength $\lambda_{e}$: The wavelength of the electronic interference signal, usually a fixed quantity reflecting the degree of interference.

Interference Frequency *f*_*e*_: The frequency of the jamming signal, which affects the effect of the noise on the system.

Interference Period *P*_*e*_: The period of the interfering signal which affects the response characteristics of the object.

Jamming Signal Strength *A*_*e*_: The strength of the electronic jamming signal, usually a fixed value to reflect the degree of jamming.

Jamming Pulse Width *T*_*e*_: The duration of the jamming effect affecting the prediction results of the object.

(2) Input matrix construction, construct the input matrix D, whose elements are the state parameters of the aircraft and ship:

$$\;D=\left[\begin{array}{cccccccccc} v_{d}(0) & h_{d}(0) & \theta_{d}(0) & \phi_{d}(0) & lat_{d}(0) & lon_{d}(0) & lat_{b}(0) & lon_{b}(0) & \psi_{s} & v_{s}\\ v_{d}(1) & h_{d}(1) & \theta_{d}(1) & \phi_{d}(1) & lat_{d}(1) & lon_{d}(1) & lat_{b}(1) & lon_{b}(1) & \psi_{s} & v_{s}\\ \vdots & \vdots & \vdots & \vdots & \vdots & \vdots & \vdots & \vdots & \vdots & \vdots \\ v_{d}(H-1) & h_{d}(H-1) & \theta_{d}(H-1) & \phi_{d}(H-1) & lat_{d}(H-1) & lon_{d}(H-1) & lat_{b}(H-1) & lon_{b}(H-1) & \psi_{s} & v_{s} \end{array}\right]$$ (1)

*H* is the number of samples.

(3) Select important features based on predefined performance metrics, such as prediction error. Set a threshold *T* to select features:

If the feature has a significant effect on the prediction (error less than the threshold), the feature is retained.

After each prediction, adjust the input features based on the current prediction error. Let the current prediction output of the model be $\hat{Y}(t)$, the true value be *Y*(*t*) and the error be.

$$E(t)=Y(t)-\hat{Y}(t)$$ (2)

Reconstruct the input matrix according to the importance of the features [26]:

$$D^{\prime }=D+\beta \cdot E(t)\cdot W$$ (3)

where $D^{\prime }$ is the reconstructed input matrix, β is the parameter controlling the intensity of the adjustment, and W is the importance weight vector reflecting the importance of each feature to the input adjustment.

(4) Volterra level modelling [27], Volterra level is used to capture the nonlinear dynamic behavior of the aircraft and the output equation can be expressed as:

$$y(t)=h_{0}+\sum_{k=1}^{n}h_{k}d(t-k)+\sum_{k_{1}=0}^{n}\sum_{k_{2}=0}^{n}h_{k_{1}k_{2}}d(t-k_{1})d(t-k_{2})+\varepsilon$$ (4)

*y*(*t*) is the output of the system at moment *t* (predicted aircraft trajectory).

*d*(*t*) is the input feature matrix.

The first-order kernel coefficient *h*_*k*_ reflects the direct effect of each feature on the output.

The second-order kernel coefficient $h_{k_{1}k_{2}}$ describes the interaction between multiple inputs, thus capturing the nonlinear characteristics of the model.

The final output equation is:

$$y(t)=h_{0}+\sum_{k=1}^{n}h_{k}d(t-k)+\frac{1}{2}\sum_{k_{1}=1}^{n}\sum_{k_{2}=1}^{n}h_{k_{1}k_{2}}d(t-k_{1})d(t-k_{2})$$ (5)

(5) Multidimensional Volterra modelling incorporating environmental factors.

$$\;{\hat{y}}_{t}=\sum_{n=0}^{N}\sum_{j=1}^{M}\int_{\tau_{1}=0}^{t}\int_{\tau_{2=0}}^{t}\cdots \int_{\tau_{n}=0}^{t}G_{n}(x_{t},x_{\tau 1},x_{\tau 2},\cdots x_{\tau n},v_{w}(\tau ),\alpha_{w}(\tau ),n(\tau ),\lambda_{e},f_{e},P_{e},A_{e},T_{e})d_{\tau 1}d_{\tau 2}\cdots d_{\tau n}+\epsilon_{t}$$ (6)

*x*_*t*_: Input feature vectors for the current moment, including object states, ship states, and environmental factors.

$x_{\tau_{i}}$: Input feature vector for the past moment, representing historical data that affects the present output.

$v_{w}(\tau )$: The wind speed at the moment.

$\alpha_{w}(\tau )$: The wind direction at the moment.

n(τ): Sensor noise at the moment.

$\lambda_{e},f_{e},P_{e},A_{e},T_{e}$: Electronic interference parameters, including wavelength, frequency, period, signal strength, and pulse width, which determine the characteristics of the object’s response to electronic interference signals.

$\epsilon_{t}$: Noise of the model output.

(6) Improved beetle tentacle search algorithm for kernel coefficients in optimization framework.

Step1: Intelligent initialization of initial tentacle positions using K-means clustering algorithm [28]:

$$x_{i}(0)=KMeans(data,clusters)$$ (7)

*x*_*i*_(0): initialized tentacle position.

Step2: The fitness of each tentacle position is evaluated by the following equation:

$$f(x_{i})=L(x_{i})=\frac{1}{M}\sum_{j=1}^{M}{\left(Y_{j}-{\hat{Y}}_{j}(x_{i})\right)}^{2}$$ (8)

*Y*_*j*_: Actual observations, ${\hat{Y}}_{j}(x_{i})$: The value predicted by the current reach position.

Step3: Optimise the tentacle position using adaptive learning rate and multi-scale search strategy, where the adaptive learning rate is:

$$\alpha (t)=\frac{\alpha_{max}}{1+\beta t}$$ (9)

Tentacle position update:

$$x_{i}(t+1)=x_{i}(t)+\alpha (t)\cdot (r(t)\cdot (x^{*}-x_{i}(t))+\sigma (t)\cdot n)$$ (10)

r(t) is a random perturbation factor, ${\text{x}}^{*}$ is the best current reach position, σ(t) is the perturbation intensity.

In the multiscale search strategy, the introduction of the temperature parameter *T*(*t*).

$$T(t)=T_{0}\cdot \exp(-\lambda t)$$ (11)

In order to enhance the global exploration capability of the search algorithm and avoid falling into local optimal solutions, larger stochastic perturbations are executed during high temperature periods.

$$x_{i}(t+1)=\left\{\begin{array}{c} \begin{array}{cc} x_{i}(t)+\alpha (t)\cdot r(t) & if\;rand<T(t) \end{array}\\ \begin{array}{cc} x^{*}+\delta & else \end{array} \end{array}\right\}$$ (12)

where $x_{i}(t)$ is the state of the tentacle position at time *t*, α(t) is the learning rate at time *t*, r(t) is the random perturbation factor, *rand* is used to decide whether to use the first update rule or the second update rule, and if its value is less than *T*(*t*) the tentacle is updated by random perturbation, *T*(*t*) is the temperature parameter at time *t*, which is used to measure the degree of openness of the search during the execution of the algorithm, the higher the degree, the more it tends to be randomly explored; at lower temperatures, the update of the tentacle position depends more on the current optimal solution ${\text{x}}^{*}$, δ being the local adaptation vector drawn randomly from the neighborhood. At higher temperatures, the tentacle position update is more dependent on the current optimal solution J, and K is a local adaptation vector drawn randomly from the neighborhood.

(7) Trajectory prediction

The model optimized by the beetle tentacle search algorithm is predicted using the optimized kernel coefficient matrix $H^{*}$.

$$\hat{Y}=H^{*}\cdot D^{\prime }$$ (13)

$H^{*}$ is the kernel coefficient matrix optimized by the ABS algorithm, $D^{\prime }$ is the input matrix adapted by the adaptive reconstruction method, and $\hat{Y}$ is the predicted aircraft trajectory output vector.

(8) Complete steps of prediction model

Step 1: Collect the speed, altitude, azimuth, pitch, position and distance of the aircraft, and the state information of the ship such as speed, heading and position, and the detection data.

Step 2: Create the input matrix D and perform feature selection and reconstruction.

Step 3: Express the aircraft motion model using Volterra series, add the ship state information, target status information as well as environmental factors.

Step4: Optimize the kernel coefficients using the improved beetle tentacle search algorithm to find the best parameter configuration to minimize the loss function.

Step5: Aircraft trajectory prediction by the optimized kernel coefficient matrix.

In summary, our proposed BAS-Volterra trajectory prediction model aims to improve the prediction accuracy and computational efficiency of aircraft manoeuvre trajectories. The improved BAS algorithm has a powerful global search capability and can effectively optimize the parameter settings of the Volterra model, thus accurately capturing the non-linear and time-varying characteristics in the time series data.

## A aircraft manoeuvre trajectory prediction model based on BAS-Volterra and adaptive ensemble learning strategy

**Fig 1:**
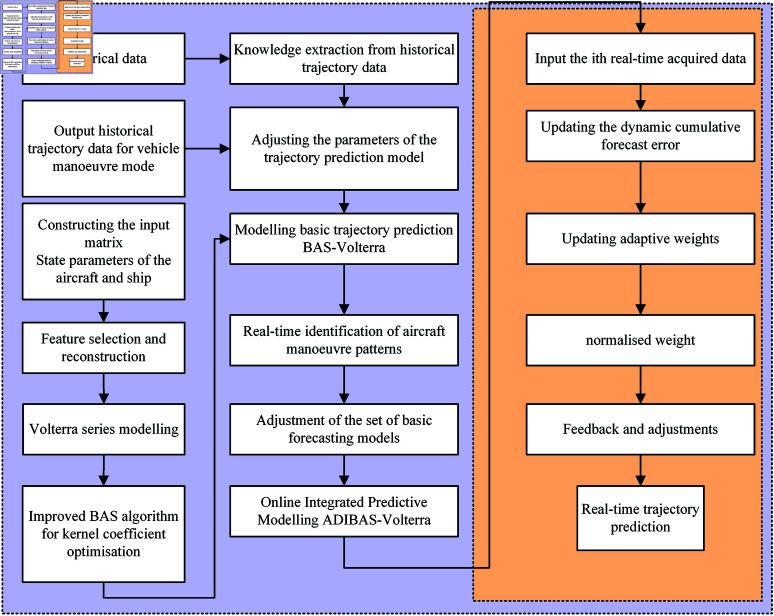
Flow chart of ADIBAS-Volterra aircraft trajectory prediction algorithm.

Ensemble learning is to use multiple basic algorithms to learn together according to the needs and combine the learning results of each basic algorithm according to certain requirements, so as to achieve better learning results than a single algorithm. In order to further enhance the accuracy of the BAS-Volterra-based aircraft manoeuvre trajectory prediction model, adaptive dynamic integration of the aircraft manoeuvre trajectory prediction model based on improved beetle tentacle search is proposed by introducing aircraft manoeuvre characteristics. The flow of our proposed ADIBAS-Volterra aircraft manoeuvre trajectory prediction model is shown in Fig 1.

### Boundary point identification model for aircraft manoeuvres

The online identification of aircraft manoeuvre patterns is a complex and dynamic process. In order to more effectively capture real-time changes in aircraft manoeuvring characteristics, this study combines the identification results of aircraft manoeuvre boundary points and dynamically adjusts the basic prediction model set to improve the prediction accuracy of aircraft manoeuvre trajectories.

The aircraft manoeuvre trajectory is made up of a series of successive manoeuvres, Thus, an aircraft’s manoeuvre trajectory can be interpreted as segments between basic manoeuvres. According to the characteristics of the different manoeuvres, the aircraft’s manoeuvres can be classified into three categories: horizontal plane manoeuvres, vertical plane manoeuvres and space manoeuvres. To better describe the different types of manoeuvres, The specific model for the aircraft manoeuvre is shown below.

$$\{\begin{array}{c} {\dot{x}}_{t}=\upsilon_{t}\cos\gamma_{t}\cos\psi_{t}\\ {\dot{y}}_{t}=\upsilon_{t}\cos\gamma_{t}\sin\psi_{t}\\ {\dot{z}}_{t}=\upsilon_{t}\sin\gamma_{t} \end{array}$$ (14)

$$\{\begin{array}{c} {\overset{.}{\nu }}_{\text{t}}=\text{g}({\text{n}}_{\text{xt}}-\sin\gamma_{\text{t}})\\ {\dot{\gamma }}_{\text{t}}=\frac{\text{g}}{{\text{v}}_{\text{t}}}({\text{n}}_{\text{zt}}\cos\varphi_{\text{t}}-\cos\gamma_{\text{t}})\\ {\dot{\psi }}_{\text{t}}=\text{g}{\text{n}}_{\text{zt}}\cos\varphi_{\text{t}}/\nu_{\text{t}}\cos\gamma_{\text{t}} \end{array}$$ (15)

Where $\left(x_{t},y_{t},z_{t}\right)$ is the position in inertial coordinates, $\overset{.}{\underset{t}{\nu }},{\dot{\gamma }}_{t},{\dot{\psi }}_{t}$ is the rate of change of the speed, pitch angle and yaw angle of the aircraft, individually, *g* is the gravitational acceleration, $\left(n_{xt},n_{zt},\varphi_{t}\right)$ is the tangential overload, normal overload and roll angle, respectively, $\left(x_{t},y_{t},z_{t},\nu_{t},\gamma_{t},\psi_{t}\right)$ and $\left(n_{xt},n_{zt},\varphi_{t}\right)$ are the state variables and control variables. According to the kinematic model of the aircraft manoeuvre trajectory, state variables and control variables, the aircraft manoeuvre trajectory can be classified into 45 manoeuvre modes, and the aircraft manoeuvre identification rules shown in Table 1 can be obtained according to the parameter settings of the 45 manoeuvre modes.

**Table 1:** Aircraft manoeuvre recognition rules.

NO.	$\psi_{t}$	$dv_{t}$	$d\gamma_{t}$	$\gamma_{t}$
A1	Left turn flight < 0	acceleration > 0	rapid climb > 0	
A2			stabilise = 0	Steady climb > 0
A3				Turn left horizontally = 0
A4				stable dive < 0
A5			dive down fast < 0	
A6		uniform speed = 0	rapid climb > 0	
A7			stabilise = 0	Steady climb > 0
A8				Turn left horizontally = 0
A9				stable dive < 0
A10			dive down fast < 0	
A11		acceleration < 0	rapid climb > 0	
A12			stabilise = 0	Steady climb > 0
A13				Turn left horizontally = 0
A14				stable dive < 0
A15			dive down fast < 0	
A16	Right turn flight > 0	acceleration > 0	rapid climb > 0	
A17			stabilise = 0	Steady climb > 0
A18				Turn left horizontally = 0
A19				stable dive < 0
A20			dive down fast < 0	
A21		uniform speed = 0	rapid climb >0	
A22			stabilise = 0	Steady climb >0
A23				Turn left horizontally =0
A24				stable dive <0
A25			dive down fast <0	
A26		acceleration < 0	rapid climb > 0	
A27			stabilise = 0	Steady climb >0
A28				Turn left horizontally = 0
A29				stable dive < 0
A30			dive down fast < 0	
A31	vertical plane motion = 0	acceleration > 0	rapid climb > 0	
A32			stabilise = 0	Steady climb > 0
A33				Turn left horizontally = 0
A34				stable dive < 0
A35			dive down fast < 0	
A36		uniform speed = 0	rapid climb > 0	
A37			stabilise =0	Steady climb > 0
A38				Turn left horizontally = 0
A39				stable dive < 0
A40			dive down fast <0	
A41		acceleration < 0	rapid climb > 0	
A42			stabilise = 0	Steady climb > 0
A43				Turn left horizontally = 0
A44				stable dive < 0
A45			dive down fast < 0	

Generally speaking, the aircraft manoeuvre trajectory can be consist of a series of manoeuvre units, based on the aircraft manoeuvre recognition rules in Table 1, the aircraft manoeuvre trajectory needs to be further segmented, as can be seen in Table 1, the characteristics of each manoeuvre unit boil down to segment rules, because of the diversity of the characteristics of the manoeuvre unit parameters, describes the conditions under which a flight manoeuvre cannot be segmented, and if these heavenly swords are not satisfied, the trajectory point at the current moment is used as the segmentation point, which can be given as follows.

$$\psi_{t-1}\cdot \psi_{t}<0\;or\;\psi_{t-1}\cdot \psi_{t}=0,\psi_{t-1}\neq 0\;or\;\psi_{t-1}\cdot \psi_{t}=0,\;\psi_{t}\neq 0$$ (16)

$${\dot{\nu }}_{t-1}\cdot {\dot{\nu }}_{t}<0\;or\;{\dot{\nu }}_{t-1}\cdot {\dot{\nu }}_{t}=0,{\dot{\nu }}_{t-1}\neq 0\;or\;{\dot{\nu }}_{t-1}\cdot {\dot{\nu }}_{t}=0,\;{\dot{\nu }}_{t}\neq 0$$ (17)

$${\dot{\gamma }}_{t-1}\cdot {\dot{\gamma }}_{t}<0\;or\;{\dot{\gamma }}_{t-1}\cdot {\dot{\gamma }}_{t}=0,{\dot{\gamma }}_{t-1}\neq 0\;or\;{\dot{\gamma }}_{t-1}\cdot {\dot{\gamma }}_{t}=0,\;{\dot{\gamma }}_{t}\neq 0$$ (18)

$${\dot{\gamma }}_{t-1}\cdot {\dot{\gamma }}_{t}<0\;or\;{\dot{\gamma }}_{t-1}\cdot {\dot{\gamma }}_{t}=0,{\dot{\gamma }}_{t-1}\neq 0\;or\;{\dot{\gamma }}_{t-1}\cdot {\dot{\gamma }}_{t}=0,\;{\dot{\gamma }}_{t}\neq 0$$ (19)

The setting of boundary conditions is very important in the manoeuvre action recognition model, and we improve the robustness and sensitivity of the model by defining multiple thresholds.

When the velocity change is less than a certain threshold, it can be regarded as a boundary point:

$$\left|\Delta v\right|<\epsilon_{v}(Velocity\;change\;less\;than\;the\;threshold)\Rightarrow The\;current\;point\;is\;regarded\;as\;the\;split\;point$$ (20)

The change in acceleration can also be used as a basis for boundary judgement:

$$\left|\Delta \alpha \right|<\epsilon_{\alpha }(\text{Acceleration changes less than threshold})\Rightarrow \text{The current point is regarded as the split point}$$ (21)

Formula update strategy under reinforcement learning model:

$$Q(s,\alpha )\leftarrow Q(s,\alpha )+\alpha [r+\gamma \max_{\alpha }Q(s^{\prime },\alpha )-Q(s,\alpha )]$$ (22)

*Q*(*s*,*a*) is the expected reward for taking an action in the state, α is the learning rate, *r* is the immediate reward, γ is the discount factor, and $s^{\prime }$ is the new state. Based on the historical learning of the model, the algorithm can continuously optimize the action selection strategy for new manoeuvre states and boundary recognition.

In terms of aircraft manoeuvre recognition, this section proposes 45 manoeuvre patterns and details the corresponding parameter settings and recognition rules, which are an important basis for manoeuvre segmentation, ensuring that the characteristics of each manoeuvre unit can be accurately recognized and that, under certain conditions, manoeuvres that cannot be segmented can still be accurately recognized.

### Model adaptive updating algorithm based on trajectory prediction performance

We constructed the ADIBAS-Volterra aircraft manoeuvre trajectory prediction model, the weights of each basic forecasting methods are referenced to the same value to construct the initial integrated prediction model, and to ensure the overall performance of the ensemble prediction model, it is necessary to adaptively update the weights of each basic predictor according to its respective prediction accuracy in the process of acquiring real-time data and the model. The rule of adaptively updating weights is that models with superior performance generally have larger weights, and some relevant definitions are listed below for ease of illustration.

$$E_{t}^{i}=y_{t}-{\hat{y}}_{t}^{i}+\lambda \sum_{j=1}^{K}\gamma^{K-j}E_{t-j}^{i}$$ (23)

where $E_{t}^{i}$ is the prediction error of the basic predictor *i* at time *t*, *y*_*t*_ is the actual aircraft value at time *t*, λ is the historical error weighting factor which controls the degree of influence of the historical error in the current prediction error, *K* is the size of the historical window which indicates the number of past prediction steps taken into account in the calculation of the current error, and γ is the damping factor which controls the degree of influence of the past error in the current prediction error, usually 0<γ<1, where the dynamic cumulative prediction error formula is shown below.

$$\omega_{t}^{i}=\omega_{t-1}^{i}\cdot (1-\alpha \cdot \frac{MSE_{t}^{i}-AMSE_{t}}{\sigma MSE_{t}+\varepsilon })+\delta \cdot \frac{E_{t}^{i}}{\frac{1}{N}\sum_{k=1}^{N}\left|E_{t}^{k}\right|}$$ (24)

$$AMSE_{t}^{i}=\frac{1}{N}\sum_{k=1}^{N}MSE_{t}^{k}$$ (25)

$\omega_{t}^{i}$ is the adaptively updated weights of the basic predictor *i* at time *t*, $\omega_{t-1}^{i}$ is the weights of the basic predictor *i* at time *t*–1, α is the learning rate, which controls the sensitivity of the weight updating, where higher values can lead to instability, and *AMSE*_*t*_ is the average dynamic cumulative prediction error of all basic predictors.

$\sigma MSE_{t}$ is the standard deviation of the dynamic cumulative prediction error of all current base predictors, which is used to quantify the stability of the prediction performance, ε is a small constant added to avoid divide-by-zero errors, which usually takes a very small value, δ is a historical error feedback factor that controls how much the current prediction error affects the weight update, and $E_{t}^{i}$ is the prediction error of the base predictors at the current time.

$$\omega_{t}=\frac{\omega_{t}^{i}}{\sum_{j=1}^{N}\omega_{t}^{j}}$$ (26)

$\omega_{t}$ is the weight of the normalized base predictor *i* at time *t*, and $\sum {\mathrm{\backslash nolimits}}_{j=1}^{N}\omega_{t}^{j}$ is the sum of the weights of all the base predictors, ensuring that the total weights are normalized to 1. We consider not only the prediction error of the model, but also the trajectory prediction performance of the model, which allows us to comprehensively evaluate the performance of the model.

## Error analysis

The experiments were conducted in the Matlab2021 environment, running on a PC with a 4-core Intel Core i9 processor and 32 GB of RAM. We use adversarial training data extracted from the simulated aircraft system as experimental data.

To better compare the performance of different algorithms, we used four measures, Relative Root Mean Square Error (RRMSE), Mean Absolute Deviation (MAD), Mean Absolute Percentage Error (MAPE) and Normalised Mean Square Error (NMSE), to evaluate the prediction accuracy, and the simulation results for each group are 100 independent experiments were averaged as follows:

$$RRMSE=\frac{\sqrt{\frac{1}{n}\sum_{t=1}^{n}{\left(\tilde{y}(k)-y(k)\right)}^{2}}}{\bar{y}}$$ (27)

$$MAD=\frac{1}{n}\sum_{t=1}^{n}\left|\tilde{y}(k)-y(k)\right|$$ (28)

$$MAPE=\frac{1}{n}\sum_{t=1}^{n}\left|\frac{\tilde{y}(k)-y(k)}{\tilde{y}(k)}\right|$$ (29)

$$NMSE=\frac{{\sum_{t=1}^{n}\left[\tilde{y}(k)-y\right]}^{2}}{{\sum_{t=1}^{n}\left[\tilde{y}(k)-\tilde{y}\right]}^{2}}$$ (30)

where $\tilde{y}$ is the actual value, *y* is the predicted value and *y* is the average value of $\tilde{y}$. To evaluate the performance of the aircraft manoeuvre trajectory prediction model based on the ADIBAS-Volterra algorithm.

### Experimental validation of aircraft manoeuvre trajectory prediction simulation

**Fig 2:**
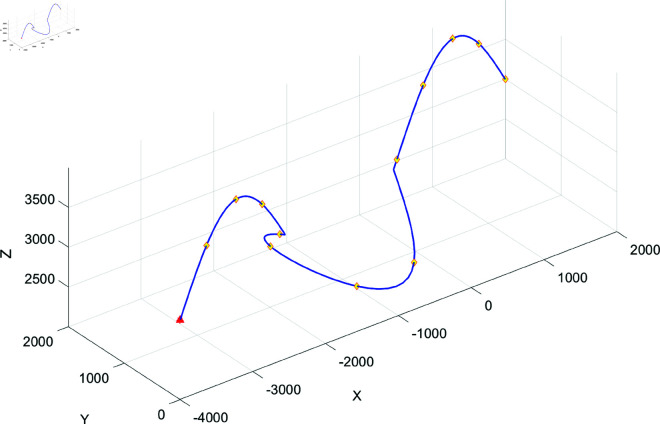
Segmentation results for one flight of the aircraft.

**Fig 3:**
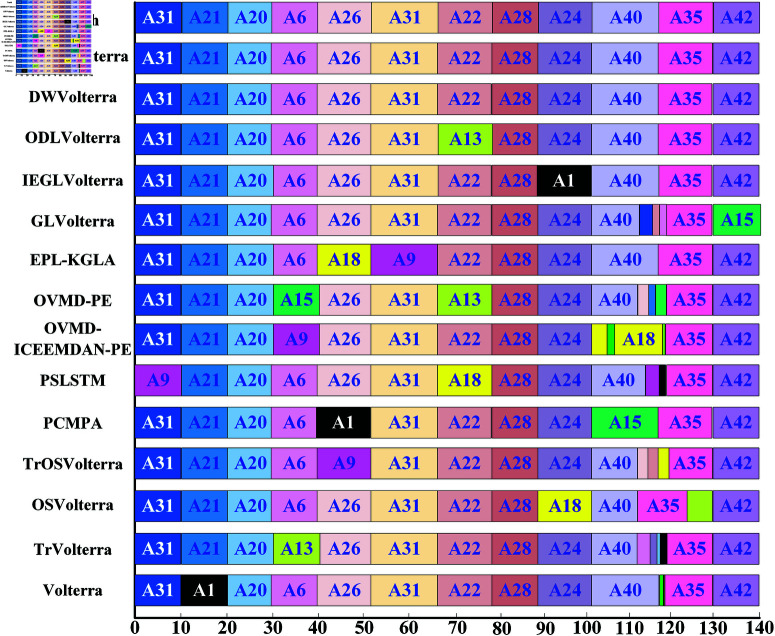
Comparison of different temporal recognition methods on a single aircraft flight manoeuvre dataset, segmentation and recognition of a long-range flight manoeuvre dataset containing 12 manoeuvre units, the black lines indicate the boundaries of the manoeuvres, the different colours used by the different methods correspond to different manoeuvres, and the bold labels written correspond to the labels of the manoeuvres.

In Fig 2, the blue trajectory indicates the flight manoeuvre of the aircraft and the orange rectangular points indicate the segmentation points. Based on the comparison of the original segmentation with the proposed segmentation, the main segmentation points correspond to the original segmentation points in terms of number and length. To further test the validity of the post-segmentation recognition, Fig 3 shows two comparisons of the recognition methods for the aircraft at different times, where the main segments are shown, with the labels of the segments in bold, and ADIBAS-Volterra correctly shows the category labels of all segments except the dependent segments. When identifying the main segments, ADIBAS-Volterra gives the correct category labels for most segments except the last one.

When identifying the main segments, ADIBAS-Volterra provided the correct category labels for all segments. Among the other methods compared, Volterra, Tr-Volterra, OS-Volterra, TrOS-Volterra, PCMPA, PSLSTM, OVMD-ICEEMDAN-PE, OVMD-PE, EPL-KGLA, GL-Volterra, IEGL-Volterra, ODL-Volterra and DW-Volterra can provide 7, 7, 6, 6, 6, 6, 6, 5, 7, 6, 7, 6, 7, 7 and 8 correct major fragments respectively. As can be seen in Fig 3, ADIBAS-Volterra and DW-Volterra can each identify all the main segments. Meanwhile, the other 11 advanced trajectory prediction methods can identify 10, 10, 10, 10, 10, 10, 10, 9, 10, 9, 10, 11 and 11 main segments respectively. In conclusion, ADIBAS-Volterra outperforms the other advanced methods in terms of segment length and correct identification of boundaries.

### Feasibility analysis of aircraft manoeuvre trajectory prediction methods

To validate the adaptability of our proposed predictive model, we used the typically chaotic time series Mackey Glass and Rossler datasets to validate the BAS-Volterra manoeuvre trajectory prediction model, synthesising the scientific validity of the improved beetle tentacle search algorithm.

To illustrate the essentiality of choosing the Volterra sequence [11] as the underlying predictor for improving the completed manoeuvre trajectory prediction model, the predictive performance of the algorithm was compared with the shallow machine learning algorithms K-means [29], (Convolutional Neural Network and Long Short-Term Memory, CNN-LSTM) [30], (Temporal Fusion Transformer and Long-Short Term Memory, TFT-LSTM) [31], Transformer [32].

The ADIBAS-Volterra prediction model is an ensemble algorithm and the improved performance of the prediction model is the outcome of improved BAS, Volterra model, online learning and adaptive ensemble learning algorithms, furthermore, in order to introduce the roles of each part of ADIBAS-Volterra, with Volterra, BAS, BAS-Volterra, improved BAS-Volterra and ADIBAS-Volterra for comparison.

In order to â€Œproveâ€Œ the superior performance of the ADIBAS-Volterra algorithm based on the aircraft manoeuvre characteristics, the model was compared with the (the Grey Lotka-Volterra model, GL-Volterra) [21], (information-enhanced Grey Lotka-Volterra model, IEGL-Volterra) [33], (one-dimensional linear Volterra-Fredholm, ODL-Volterra) [34], (Daubechies wavelets-Volterra, WV-Volterra) [35] for comparison, aircraft manoeuvre trajectory prediction based on a single learning model belongs to the global modelling approach, which is complex and prone to local optima. Utilising multiple learning algorithms tends to achieve higher accuracy than a single learning algorithm, and in recent years, the cooperation of multiple learning algorithms has been increasingly applied to trajectory prediction problems.

In order to better compare the scientific validity of the BAS-Volterra algorithm, we compared the feasibility of the algorithm for different datasets by adding noise to the simulation. Chaotic time datasets are typically used to test the performance of nonlinear systems. We used typical column Mackey and Rossler datasets as algorithm training samples and test samples to validate the effectiveness of the algorithm, and the data generation conditions were exactly the same as those of the reference [36] are fully consistent. In order to validate the effectiveness and advancement of our proposed algorithm, we used (The paper culminates in presenting an enhanced version of the Marine Predator Algorithm, PCMPA) [37], (a partial least squares based pruning algorithm is hereby proposed for a simplified LSTM, PSLSTM) [38], (optimal variational mode decomposition, improved complete ensemble empirical mode decomposition and permutation entropy, OVMD-ICEEMDAN-PE) [39], (optimal variational mode decomposition with permutation entropy, OVMD-PE) [40], (kernel general loss algorithm based on evolving participatory learning, EPL-KGLA) [41] for comparison. A 15% noise is added to all samples of the two data sets. Noise is added as follows [42]: the standard deviation of each dimension of the test sample data is calculated, random numbers satisfying the distribution are generated and superimposed on the corresponding dimension of the sample data.

Table 2 shows the prediction results for the Mackey and Rossler datasets. In terms of the prediction accuracy of the algorithm, the RRMSE, MAD, MAPE and NMSE of ADIBAS-Volterra are much lower than those of the other methods, and the prediction accuracy of the algorithm is notably better than that of the other state-of-the-art prediction algorithms under the setting of the same test dataset, hardware and software, and parameter settings. In terms of algorithm runtime, ADIBAS-Volterra is more time-efficient than the other five prediction algorithms because Volterra treats the relationship between the time variables and the system state in the form of a convolution, which reduces the time required for model creation and tuning, and the BAS accelerates parameter convergence, allowing the model to achieve better prediction performance in a shorter time. The model also introduces an online learning mechanism, which allows the model to adjust its parameters in real time after receiving new data, and this adaptive ability can automatically optimize the model according to the changes in real-time data, thus reducing the time needed to adjust the model when a new situation arises, the runtime of the algorithm shows that the runtime of the improved ADIBAS-Volterra manoeuvre trajectory prediction model can meet the real-time requirements.

Table 2 lists the trajectory prediction results for the Mackey and Rossler datasets with noise, and it can be seen that the BAS-Volterra prediction algorithm has a high trajectory prediction accuracy with the addition of noise, and its feasibility is significantly better than that of other advanced trajectory prediction methods. By comparing the prediction results of PCMPA, PSLSTM, OVMD-ICEEMDAN-PE, OVMD-PE and EPL-KGLA algorithms, the scientific validity of our proposed algorithms is verified, and our proposed method can perform trajectory prediction efficiently under noisy conditions, which improves the prediction accuracy of the algorithm.

**Table 2:** One-step prediction results for the trajectory prediction dataset with added noise.

chaotic time series (math.)	Prediction algorithms	RRMSE	MAD	MAPE	NMSE	PTAS/s	PTPS/s
MackeyGlass	PCMPA	0.1379	0.0786	0.0715	0.1547	0.0709	6.5743E5
Rossler	PCMPA	3.5922	1.8769	1.7983	0.3562	0.0485	1.4652E4
MackeyGlass	PSLSTM	0.0890	0.0495	0.0640	0.1683	0.0586	7.5920E5
Rossler	PSLSTM	2.4694	1.3682	1.5643	0.3452	0.0572	1.5877E4
MackeyGlass	OVMD-ICEEMDAN-PE	0.1236	0.0673	0.0662	0.1726	0.0768	6.4503E5
Rossler	OVMD-ICEEMDAN-PE	2.7849	1.4592	0.9784	0.3089	0.0679	1.7688E4
MackeyGlass	OVMD-PE	0.0991	0.0230	0.0452	0.1264	21.560	2.57624E3
Rossler	OVMD-PE	2.9562	1.6979	0.6967	0.2350	0.0968	2.4539E4
MackeyGlass	EPL-KGLA	0.0857	0.0583	0.0493	0.1199	5.7694	5.5968E2
Rossler	EPL-KGLA	2.4785	1.4276	0.8750	0.2968	0.0699	1.9377E4
MackeyGlass	BAS-Volterra	0.0648	0.0452	0.0326	0.0991	0.6182	6.3542E4
Rossler	BAS-Volterra	2.0362	1.0233	0.5599	0.1946	0.3593	7.5458E4
MackeyGlass	ADIBAS-Volterra	0.0369	0.0341	0.0310	0.0891	0.6052	7.1596E4
Rossler	ADIBAS-Volterra	0.0965	1.0027	0.3132	0.1763	0.3220	8.7942E4

**Fig 4:**
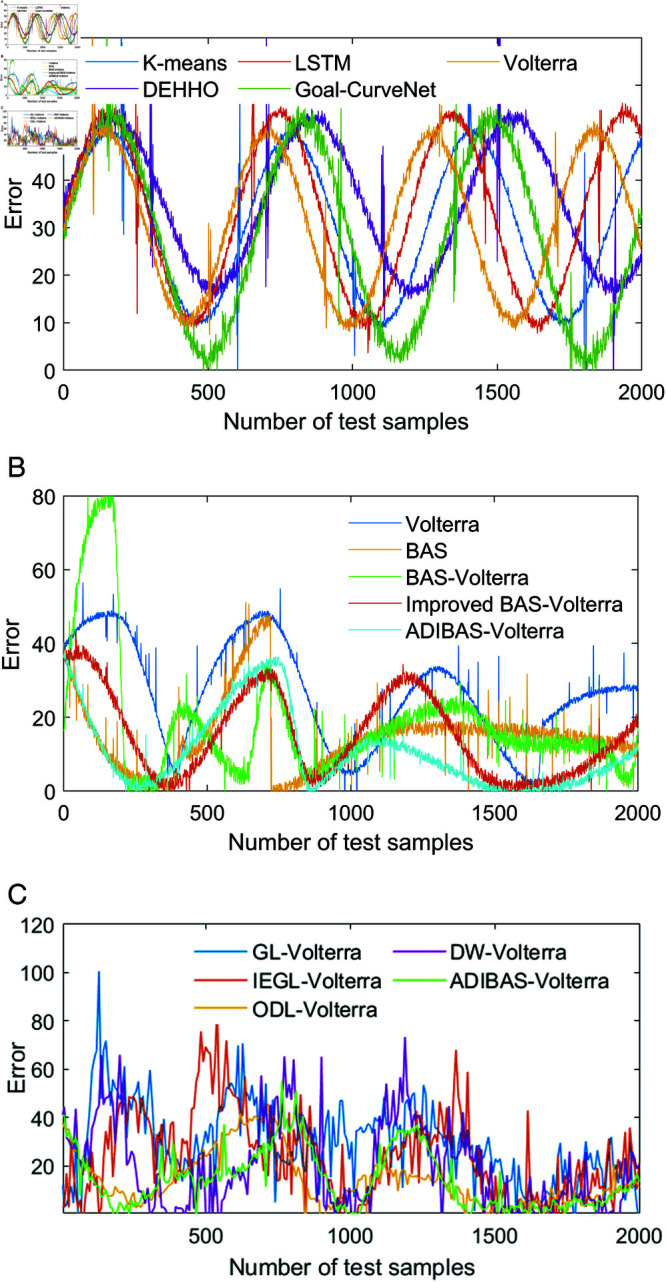
One-step trajectory prediction results for the X-coordinate: (a) one-step prediction error for the basic single algorithm; (b) one-step prediction error for each single algorithm in the algorithm; (c) one-step prediction error comparison for the ensemble prediction algorithm.

In order to prove the scientific validity of our proposed aircraft prediction model, the prediction performance of the algorithms was tested based on the aircraft manoeuvre trajectory data stored in the simulator. The parameter settings of the algorithms are shown in Table 3, and the parameter settings of the other comparison algorithms are the same as those in the literature. In order to â€Œdecrease the random errors of the prediction algorithms, the prediction results of the algorithms are the average of the results of the 100 repetitions of the experiments, and the one-step prediction results of the different algorithms on the manoeuvring trajectories of the aircraft are shown in Tables 4 to 6. The one-step prediction results of different algorithms for the aircraft manoeuvre trajectory are shown in Table 4 to Table 6. In order to compare the accuracy of different algorithms for the aircraft manoeuvre trajectory more intuitively, the one-step absolute prediction errors of different algorithms for the X, Y and Z coordinates are shown in ++Fig 4 to Fig 6.

**Fig 5:**
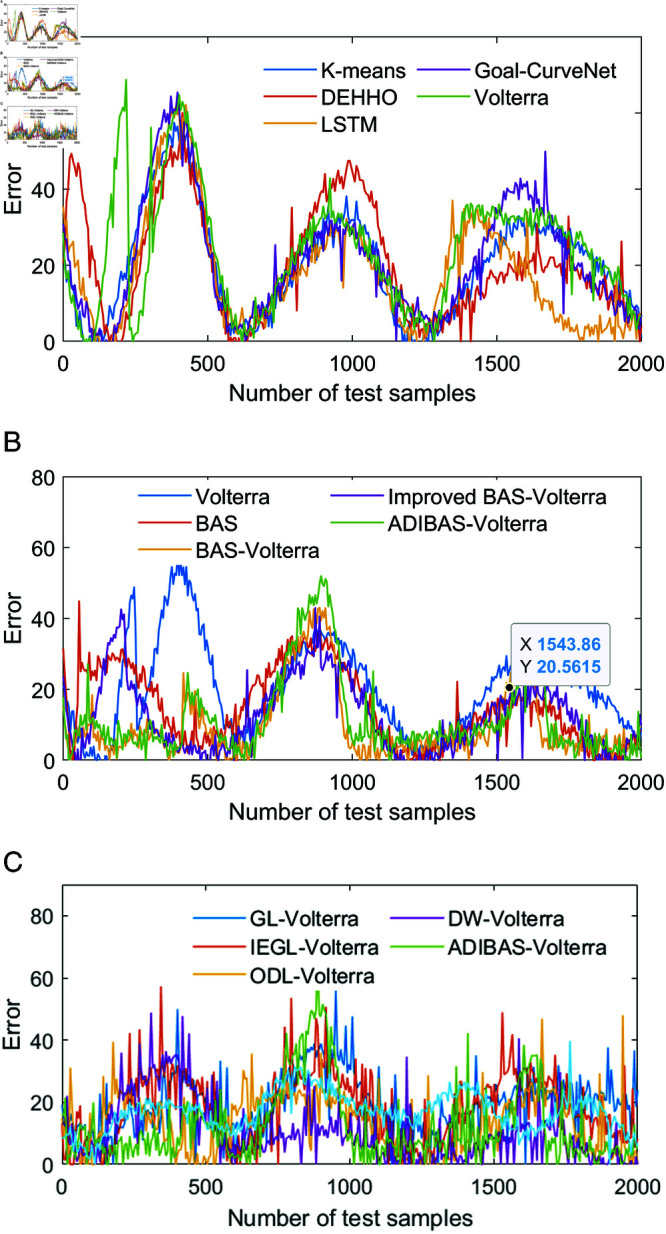
One-step trajectory prediction results for the Y-coordinate: (a) one-step prediction error for the basic single algorithm; (b) one-step prediction error for each single algorithm in the algorithm; (c) one-step prediction error comparison for the ensemble prediction algorithm.

**Fig 6:**
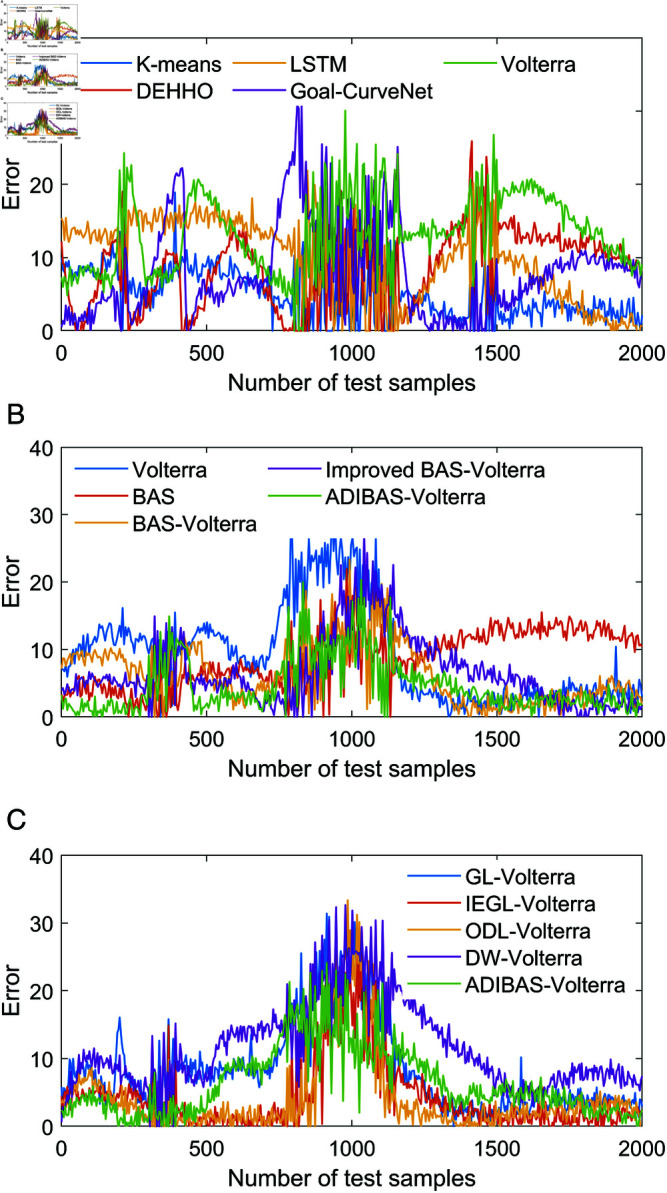
One-step trajectory prediction results for the Z-coordinate: (a) one-step prediction error for the basic single algorithm; (b) one-step prediction error for each single algorithm in the algorithm; (c) one-step prediction error comparison for the ensemble prediction algorithm.

**Table 3:** Algorithm and its parameter settings.

algorithm	Algorithm Parameter Setting
Volterra	*q* = 2
BAS	q=2,C=20
BAS-Volterra	q=2,Block=20
Improved BAS-Volterra	q=2,C=20,Block=20
GL-Volterra	*q* = 2
IEGL-Volterra	q=2,C=20
ODL-Volterra	q=2,Block=20
DW-Volterra	q=2,Block=20
ADIBAS-Volterra	$q=2,\delta =20,Block=20,M_{\min}=10,M_{\max}=20$

**Table 4:** One-step prediction results for the X-coordinate.

x-coordinate	Prediction algorithms	RRMSE	MAD	MAPE	NMSE	PTAS/s	PTPS/s
One-step prediction erroreak of the basic predictor	K-means	23.7980	18.3552	6.4399E4	6.3552E4	1.0585	6.1431E4
	CNN-LSTM	20.3752	16.7913	5.3159E4	4.7656E4	1.4730	6.2877E4
	TFT-LSTM	24.7973	25.3870	8.9630E4	0.0039	0.5972	5.4352E4
	Transformer	28.4664	22.4140	7.5474E4	0.0028	36.4528	3.5822E4
	Volterra	19.5873	14.6332	4.6078E4	2.8926E4	1.5246	6.7930E4
One-step prediction erroreak for each independenteak component	Volterra	19.3796	14.2246	4.1715E4	2.6560E4	1.5990	6.8601E4
	BAS	26.4552	26.4768	7.3568E4	0.0044	33.5231	2.4236E3
	BAS-Volterra	23.1650	24.3969	6.4262E4	0.0056	32.6443	5.6903E3
	improved BAS-Volterra	19.7561	20.5753	2.3689E4	0.0027	19.6572	2.6471E2
	ADIBAS-Volterra	9.7598	7.4820	1.5679E4	1.3861 E4	10.8471	6.2890E3
One-step prediction erroreak of ensemble predictioneak algorithms	GL-Volterra	16.2796	18.3998	3.4528E4	1.9854E4	9.5762	4.9358E3
	IEGL-Volterra	13.4588	17.4646	3.9876E4	2.4590E4	21.4688	5.5890E3
	ODL-Volterra	16.6430	16.3858	2.5570E4	1.9963E4	7.4892	2.6871E2
	DW-Volterra	19.3552	14.2568	2.8798E4	2.3466E4	12.8972	5.8962E3
	ADIBAS-Volterra	10.5685	8.5260	2.0048E4	1.2668E4	10.9990	6.9990E3

**Table 5:** One-step prediction results for Y-coordinate.

y-coordinate	Prediction algorithms	RRMSE	MAD	MAPE	NMSE	PTAS/s	PTPS/s
One-step prediction erroreak of the basic predictor	K-means	23.8560	20.6732	0.0214	5.4652E4	1.5276	6.7604E4
	CNN-LSTM	19.4531	18.8041	0.0095	4.7433E4	1.6933	9.4922E4
	TFT-LSTM	17.5289	16.4362	0.0071	3.6789E4	1.5682	7.4058E4
	Transformer	15.2006	14.5709	0.0059	1.6492E4	1.9679	6.5277E4
	Volterra	16.5732	17.5026	0.0301	5.4869E4	20.3682	5.8159E3
One-step prediction erroreak for each independenteak component	Volterra	11.4633	19.4829	0.0186	6.4539E4	0.0568	3.8544E2
	BAS	18.5762	10.6208	0.0130	5.4671E4	17.8562	6.3690E3
	BAS-Volterra	16.4298	16.8692	0.0084	6.8260E4	13.5602	5.2486E2
	improved BAS-Volterra	14.3601	11.8571	0.0065	8.8903E4	10.4680	8.5248E3
	ADIBAS-Volterra	10.6894	6.2089	0.0038	3.5622E4	10.2692	5.3562E3
One-step prediction erroreak of ensemble predictioneak algorithms	GL-Volterra	12.5682	9.8640	0.0082	1.5582E4	11.3569	8.7511E3
	IEGL-Volterra	13.6841	10.4892	0.0096	2.6731E4	10.7504	9.4472E3
	ODL-Volterra	15.3927	9.5668	0.0090	3.5962E4	12.4822	8.7803E3
	DW-Volterra	12.6870	11.5731	0.0072	1.8971E4	13.9067	7.4659E3
	ADIBAS-Volterra	10.5942	5.9952	0.0046	1.8652E4	9.4721	5.3920E3

**Table 6:** One-step prediction results for Z-coordinate.

z-coordinate	Prediction algorithms	RRMSE	MAD	MAPE	NMSE	PTAS/s	PTPS/s
One-step prediction erroreak of the basic predictor	K-means	9.4526	6.4196	0.0036	1.1347E4	2.9479	1.3651E3
	CNN-LSTM	8.2268	6.0384	0.0024E4	8.9456E5	2.4988	1.2873E3
	TFT-LSTM	6.8429	4.5892	8.7922	7.6526E5	2.0021	1.1689E3
	Transformer	5.3973	4.6921	8.4257E4	5.1992E5	2.0428	1.0086E3
	Volterra	6.4527	4.4342	0.0089	1.8864E4	1.3350	6.8821E2
One-step prediction erroreak for each independenteak component	Volterra	5.3905	5.5871	0.0078	2.4268E4	12.5627	3.9942E4
	BAS	7.4517	5.7956	0.0059	2.0986E4	10.1374	9.8730E5
	BAS-Volterra	6.5633	4.6862	0.0072	2.4698E4	9.5002	7.0745E2
	Improved BAS-Volterra	5.3924	3.9042	0.0058	4.5892E4	9.6713	3.9048E3
	ADIBAS-Volterra	3.1583	2.2039	4.0139E4	1.8266E5	10.9271	6.4572E3
One-step prediction erroreak of ensemble predictioneak algorithms	GL-Volterra	6.7862	5.3670	5.5279E4	4.7896E5	9.5219	9.5763E3
	IEGL-Volterra	5.4933	2.7859	4.3621E4	4.9859E5	10.6126	5.6892E3
	ODL-Volterra	4.9998	4.0988	8.8062E4	6.0621E5	11.6682	6.5941E3
	DW-Volterra	5.0396	3.6144	6.6690E4	4.4230E5	10.8226	5.6895E3
	ADIBAS-Volterra	4.9684	2.5531	5.6051E4	3.6672E5	11.9954	7.9990E3

The RRMSE, MAD, MAPE, NMSE, processing time for all samples (PTAS) and processing time per sample (PTPS) for the different algorithms are shown in Tables 4 to 6. In addition, the absolute prediction error data of all the above methods are plotted to visually compare the performance of the different methods. From the prediction error data We summarize below:

(1) According to the comparison of one-step prediction errors of Volterra model in X coordinate, Y coordinate and Z coordinate, as well as the prediction indexes of Kmeans, CNN-LSTM, TFT-LSTM, Transformer, Volterra as shown in Fig 4 to Fig 6, it can be seen that the one-step prediction performance of Volterra model is better than that of the other prediction methods, and the prediction time meets the real-time requirement of the algorithm. The prediction time meets the real-time requirement of the algorithm. In addition, combining the performance evaluation indexes of X-coordinate, Y-coordinate and Z-coordinate shown in Figs 4-6, it can be seen that the prediction performance of Volterra sequence is more reasonable than the other four basic prediction algorithms, and the real-time performance of Goal-Curve Net is the best, but the fluctuation of its prediction value is very large, which is unfavourable to decision-making.

(2) Comparing the prediction performance indexes of Volterra, BAS, BAS-Volterra, Improved BAS-Volterra and ADIBAS-Volterra, combined with the absolute errors shown in Figs 4~6, it can be seen that the beetle tentacle search algorithm, the aircraft manoeuvre boundary point identification algorithm and the adaptive updating weight strategy are conducive to the improvement of the algorithm. The aircraft manoeuvre boundary point identification algorithm improves the adaptability of the algorithm to changing environments and complex scenes by dynamically identifying and adjusting the boundaries of moving aircrafts. The adaptive updating weight strategy, on the other hand, dynamically adjusts the weight parameters in the algorithm by analysing the historical data and the current state in real time, allowing the model to adapt more flexibly to different data distributions and features.

(3) Comparing the trajectory prediction indexes of GL-Volterra, IEGL-Volterra, ODL-Volterra, DW-Volterra and ADIBAS-Volterra, it can be seen that the one-step prediction performance of ADIBAS-Volterra is the best, which is notably better than the prediction results of other multi-algorithm integrations, Aircraft-based The improved multi-algorithm based on the aircraft manoeuvre boundary point identification algorithm can significantly improve the prediction performance and more effectively find the changes in the time series feature parameters of the aircraft manoeuvre trajectory, so as to achieve the real-time updating of the parameters, and further improve the scientific validity of the prediction.

## Conclusion

We propose an adaptive hybrid algorithm based on a beetle tentacle search algorithm, the aircraft manoeuvre boundary point identification algorithm and Volterra sequences, called ADIBAS-Volterra, for time series prediction of aircraft manoeuvre trajectories. Prediction accuracy is improved by the Volterra model, which captures the non-linear and time-varying characteristics and uses the convolutional form to deal with the complex relationship between the time variables and the system state. Meanwhile, the improved Beetle Tentacle Search Algorithm (ABS) is used to enhance the global search capability of the model and avoid the local optimal solution problem, which improves the robustness and real-time responsiveness in complex environments. The RRMSE, MAD, MAPE and NMSE of ADIBAS-Volterra are much lower than those of other methods, and the single-step prediction errors of the single-step prediction ensemble prediction algorithm in X coordinate are RRMSE 10.5674, MAD 8.5249, MAPE 0.0002 and NMSE 0.0001, respectively, and the single-step prediction errors of the single-step prediction ensemble prediction algorithm in Y coordinate are RRMSE 10.4982, MAD 5.8946, MAPE 0.0039 and NMSE 0.0002, respectively. prediction error for the single-step prediction ensemble prediction algorithm in Y-coordinate is RRMSE 10.4982, MAD 5.8946, MAPE 0.0039, and NMSE 0.0002, respectively, and the single-step prediction error for the single-step prediction ensemble prediction algorithm in Z-coordinate is RRMSE 4.9562, MAD 2.5401, MAPE 0.0006, and NMSE 0.0004, respectively, which indicates the scientific validity, the novelty of the trajectory prediction model can be summarized as follows:

(1) Since the aircraft manoeuvre trajectory prediction is a time-series problem affected by a variety of nonlinear factors and dynamic environments, the Volterra model is introduced. The Volterra model is able to effectively capture the nonlinear characteristics of the system, and it is suitable for predicting the behavior of the complex dynamic system. The model, through the form of polynomial convolution, is able to deal with the changes of the time variables and adapts to the different time points of the dynamic characteristics. This method can take into account different types of disturbances more comprehensively and improve the reliability of prediction.

(2) To compensate for the limitations of a single Volterra model in capturing the nonlinear dynamic characteristics of aircraft manoeuvre trajectories, a beetle tentacle search framework suitable for aircraft manoeuvre trajectory prediction is constructed. The method optimises the model parameters by ABS, uses intelligent initialisation and multi-scale search strategies to effectively enhance the global search capability, avoids the problem of local optimal solutions, and improves the real-time online prediction performance of the model.

(3) By combining the aircraft manoeuvre boundary point identification algorithm and the prediction performance, the adaptive update integrated prediction model is constructed. In order to better adapt to the aircraft manoeuvre trajectory prediction problem, the ADIBAS-Volterra prediction model combines the aircraft manoeuvre boundary point identification algorithm with the prediction performance and performs real-time dynamic updating of the integrated model, and at the same time, based on the prediction performance indexes of the basic predictor, the weights are dynamically assigned to ensure the effectiveness and accuracy of the integrated model.

The ADIBAS-Volterra algorithm enhances the global search capability of the model and avoids the local optimal solution problem by effectively capturing the non-linear and time-varying characteristics. Meanwhile, with the dynamic update strategy and adaptive weighting, ADIBAS-Volterra significantly improves the accuracy and robustness of the prediction and adapts to the requirements of trajectory prediction in complex dynamic environments.

Feasibility of ADIBAS-Volterra model implementation in high-speed scenarios with real-time, accuracy and robustness, the combination of improved beetle tentacle search algorithm and Volterra model enables the model to quickly adapt to dynamic changes and maintain high prediction accuracy, and adaptive reconfiguration method reduces the influence of noise and enhances the stability of the model in complex dynamic environments. Meanwhile, the Volterra model in the form of convolution achieves efficient computation and meets the demand for real-time prediction. Although challenges such as data acquisition delay and external interference may be faced in practical applications, these problems can be solved by optimizing the acquisition system and interference suppression techniques, so the ADIBAS-Volterra model has the potential to play an important role in high-speed trajectory prediction, and its application prospects will be broader with the advancement of technology.
